# Supplementary figures and images for: Genomic analysis of 61 Chlamydia psittaci strains reveals extensive divergence associated with host preference
Source: BMC Genomics. 2023 May 29;24:288. doi: 10.1186/s12864-023-09370-w (PMC10226258; doi:10.1186/s12864-023-09370-w)

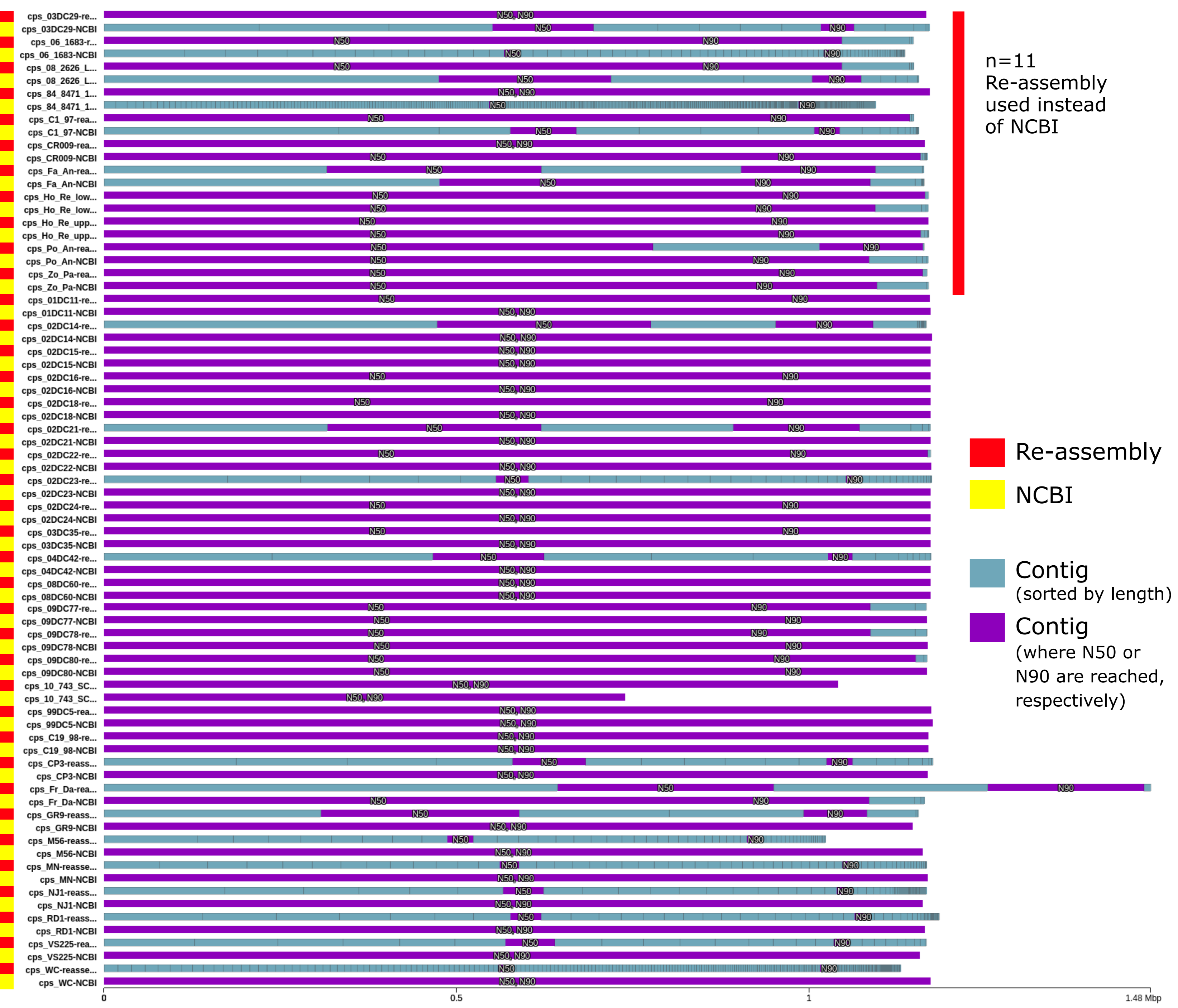

Supplement: Supplementary file 1 — Additional file 1: Figure S1. Assembly contiguity and size of 38 re-assembled genomes. For n = 38 NCBI genomes we were able to find Illumina short-read sequencing data to reassemble these strains. The Icarus plot (output of Quast v5.2.0 run with default parameters) shows the assembly contiguity and size for all 38 re-assemblies together with the corresponding genomes downloaded from NCBI. In addition, re-assemblies and NCBI genomes were decontaminated using CLEAN. The top eleven re-assemblies were finally selected to be integrated in our study and replaced the original NCBI genomes due to higher assembly contiguity and better N50 values. All contigs are sorted by length, starting with the longest contigs on the left and decreasing in length to the right. Thus, it is possible to mark the contigs where 50% (90%) of all the nucleotides in an assembly are covered by this contig and all longer contigs as a measure of assembly contiguity and quality. The purple bars mark contigs where a certain Nx (N50 or N90) is reached in an assembly. [file 12864_2023_9370_MOESM1_ESM.pdf]

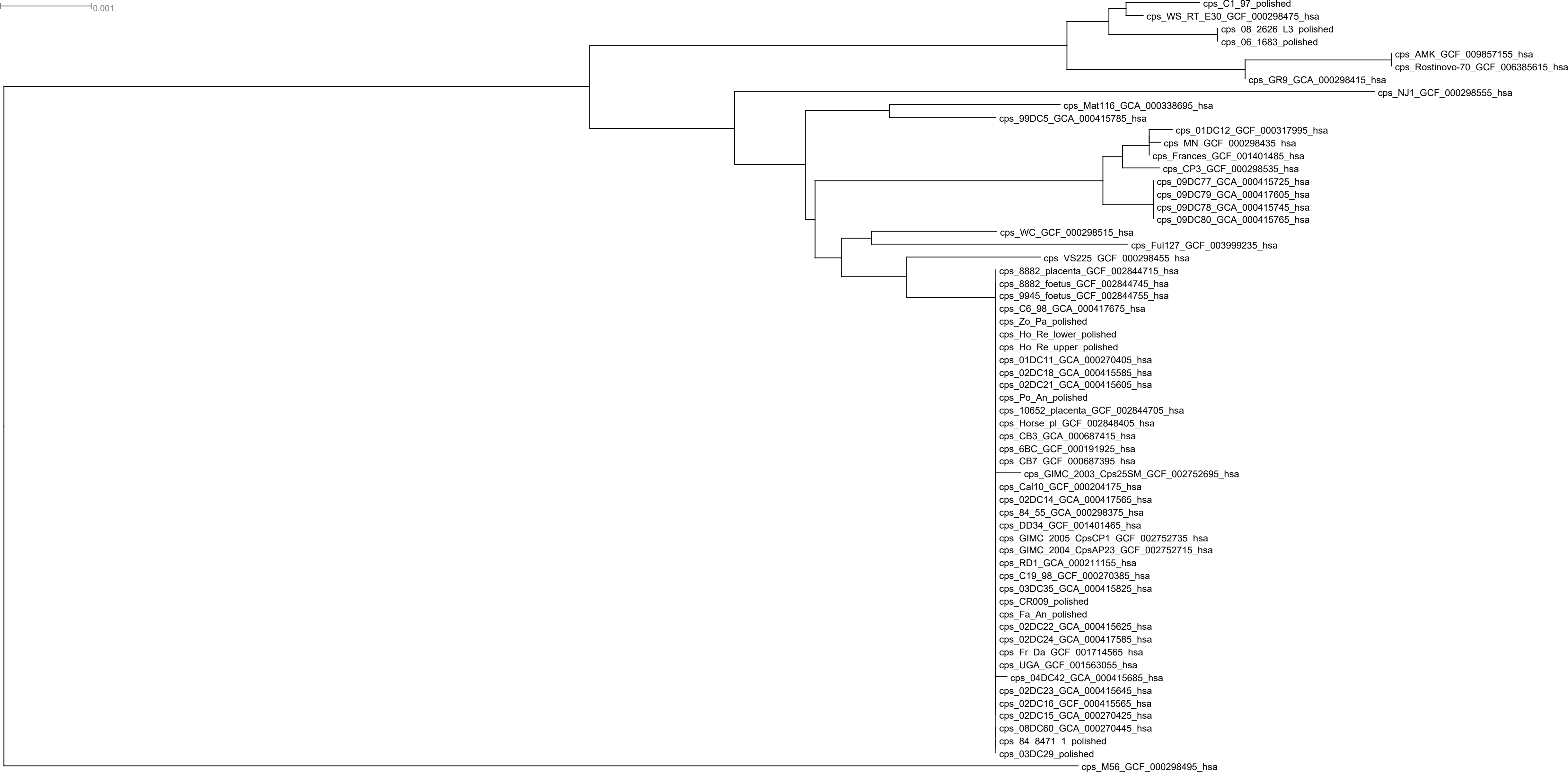

Supplement: Supplementary file 2 — Additional file 2: Figure S2. Phylogenetic tree based on the 904 common genes of 61 C. psittaci strains. The tree was reconstructed based on the concatenated core gene alignments at protein level produced by RIBAP and calculated using FastTree. [file 12864_2023_9370_MOESM2_ESM.pdf]

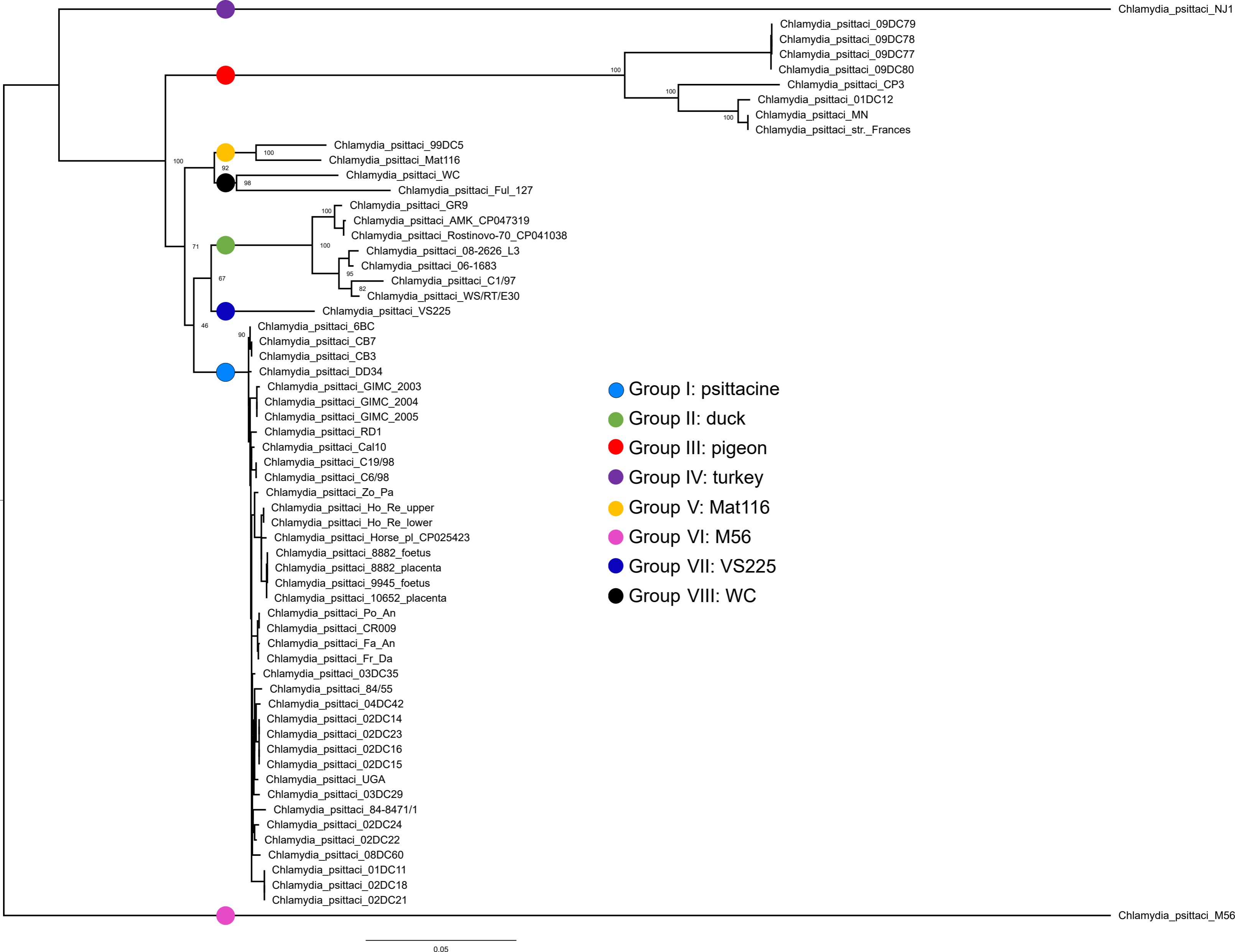

Supplement: Supplementary file 3 — Additional file 3: Figure S3. SNP-based tree determined from 61 C. psittaci genomes. The eight distinct lineages defined by Vorimore et al. [37],i.e. group I_psittacine, group II_duck, group III_pigeon, group IV_turkey,group V_Mat116, group VI_M56, group VII_VS225, and group VIII_WC, are represented by colored circles. The tree was built using RAxML version 8.2.9 with the GTRGAMMA model and 1000 bootstrap replicates based on thefiltered SNP matrix (4011 SNPs) from BioNumerics. [file 12864_2023_9370_MOESM3_ESM.pdf]

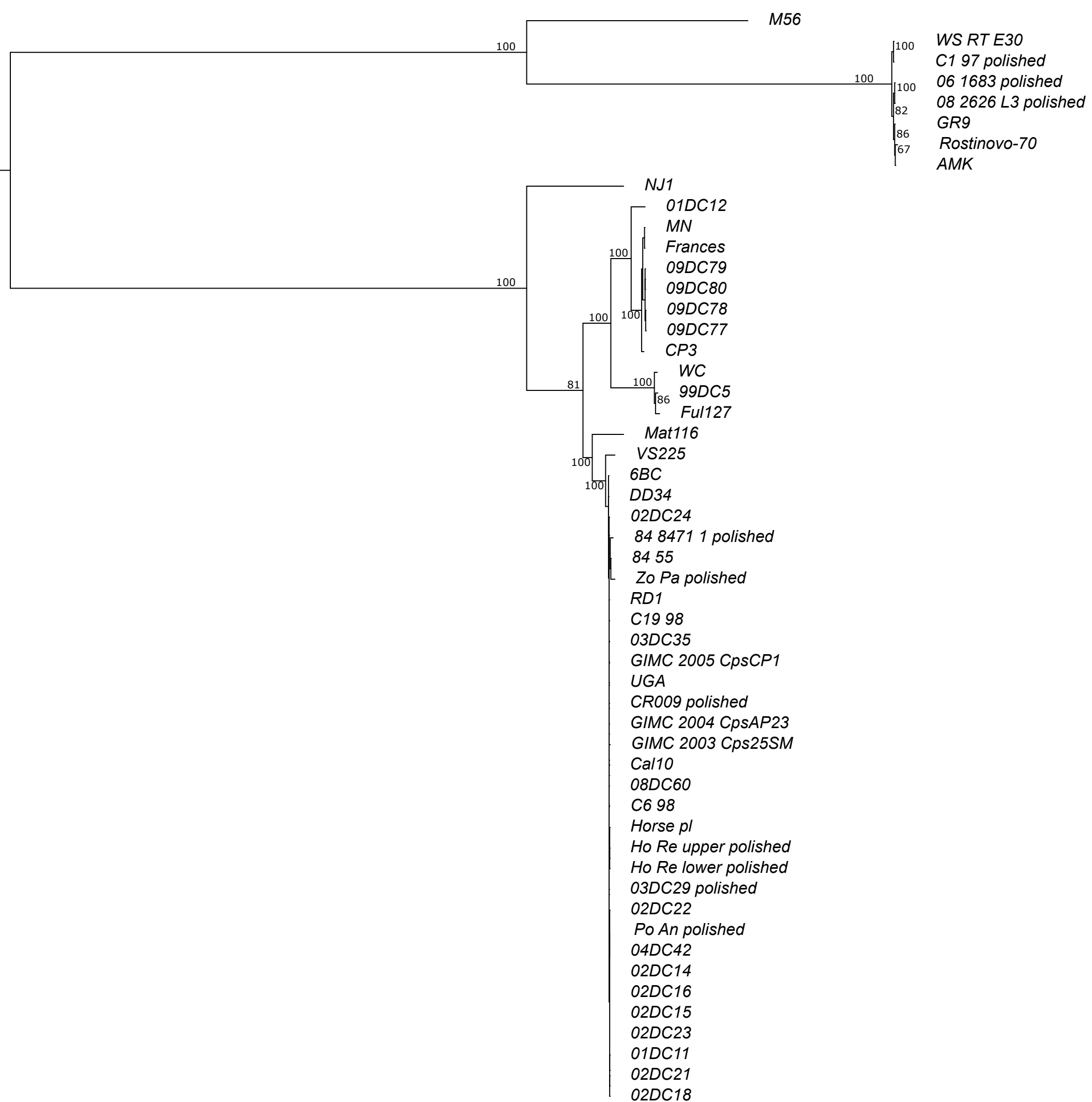

substitutions/site

0.05

0.1

0.15

Supplement: Supplementary file 4 — Additional file 4: Figure S4. Phylogenetic tree based on nucleotide sequences of the extracted PZ of 53 C. psittaci strains used in this study. Sequences of 8 strains, where this region was located on several scaffolds, were not included here. The tree was constructed using RAxML v8.2.11 with GTRGAMMA nucleotide model and Rapid hill-climbing algorithm. [file 12864_2023_9370_MOESM4_ESM.pdf]

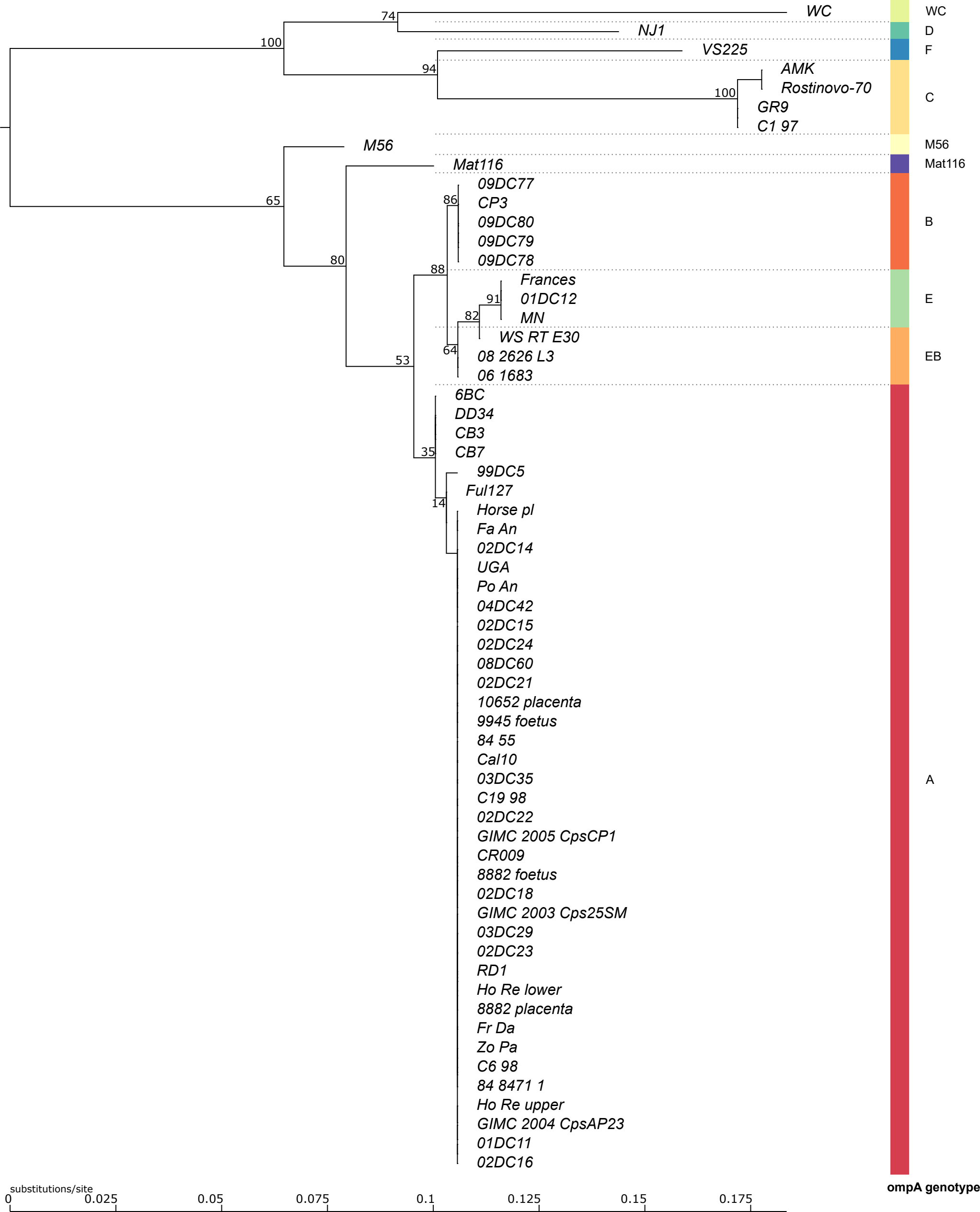

Supplement: Supplementary file 5 — Additional file 5: Figure S5. RAxML tree of the alignment of OmpA amino acid sequences from 61 C. psittaci strains as processed by RIBAP (Group 879). Bootstrap values are indicated at inner nodes. For identical taxa, bootstrap values were discarded, due to the interchangeability of corresponding gene sequences. The colored bar on the right indicates the respective ompA genotypes. [file 12864_2023_9370_MOESM5_ESM.pdf]

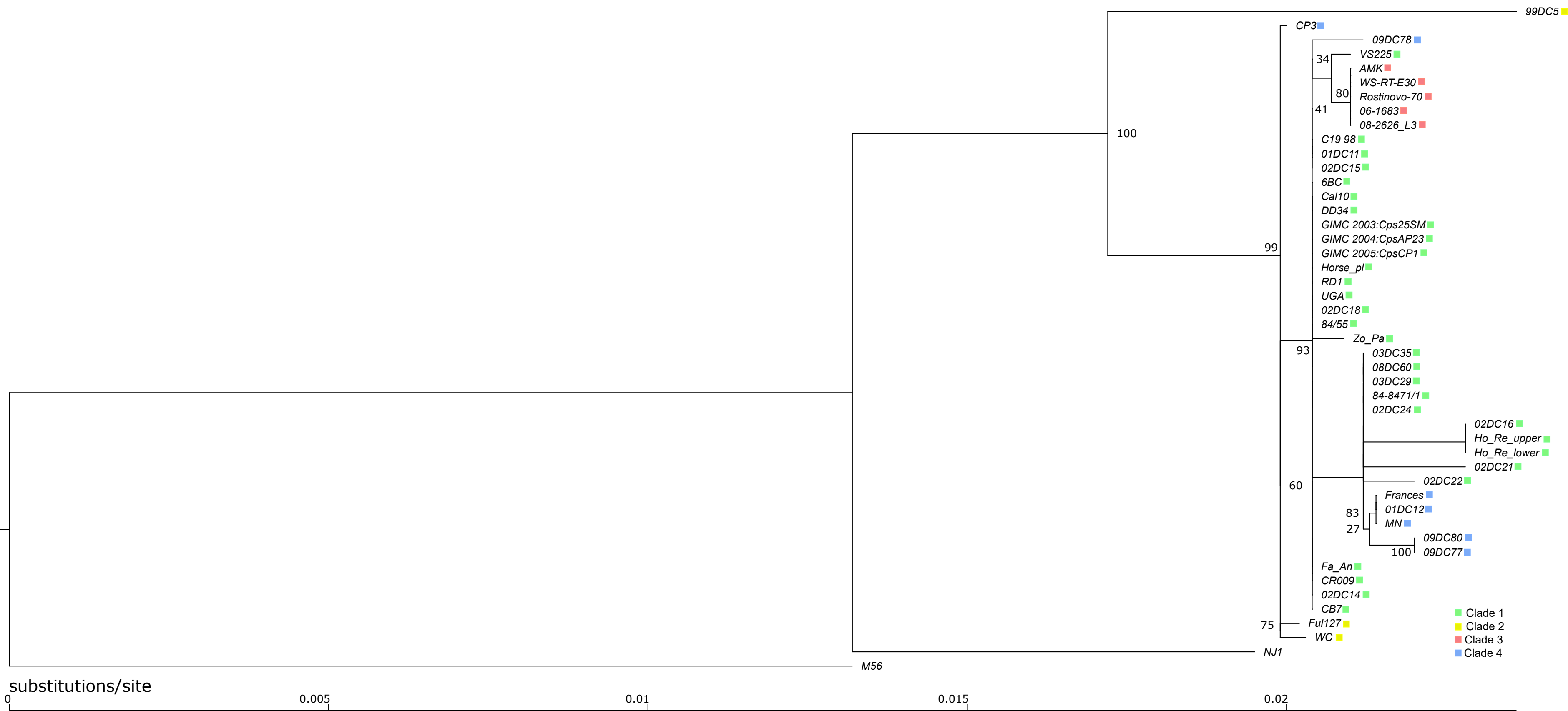

Supplement: Supplementary file 6 — Additional file 6: Figure S6. Phylogenetic tree reconstructed from the alignment of re-assembled and re-arranged plasmid sequences from 47 C. psittaci strains. The tree was built using RAxML version 8.2.12 with the GTRGAMMA model and 1000 bootstrap replicates. [file 12864_2023_9370_MOESM6_ESM.pdf]

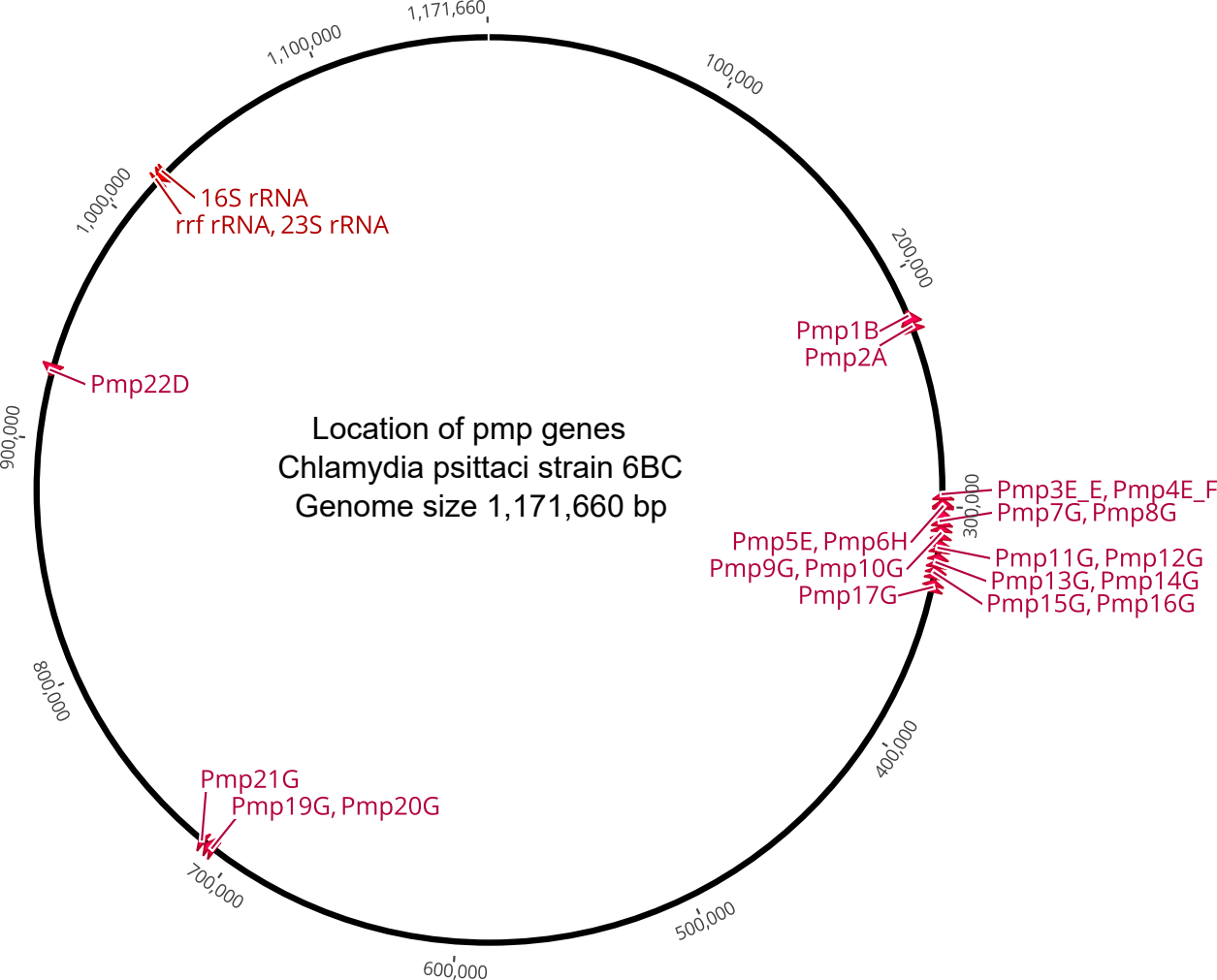

Supplement: Supplementary file 7 — Additional file 7: Figure S7. Location of genes encoding polymorphic membrane proteins in the genome of C. psittaci strain 6BC. [file 12864_2023_9370_MOESM7_ESM.pdf]

**A**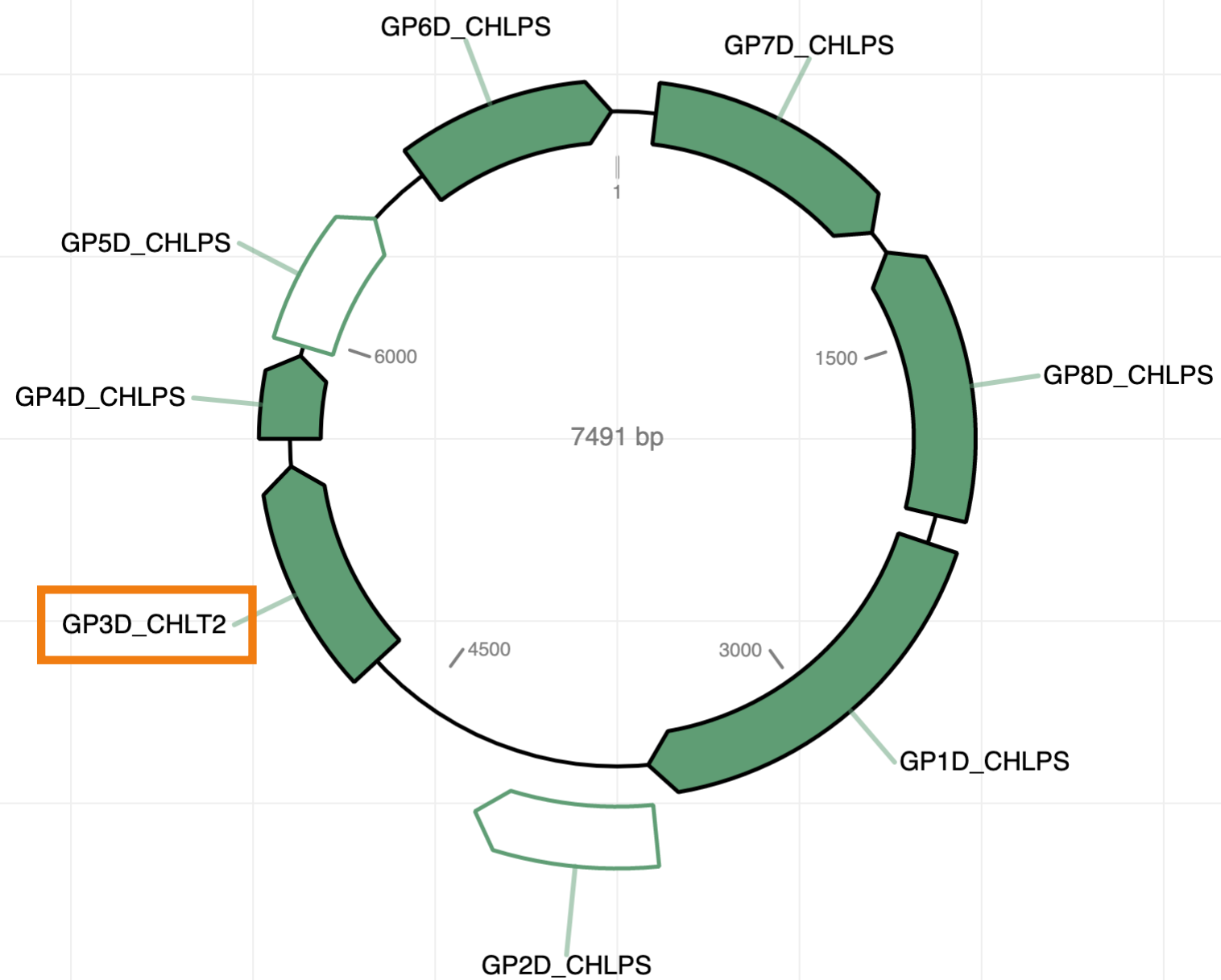

■ CDS

*C. psittaci* MN, NCBI

**B**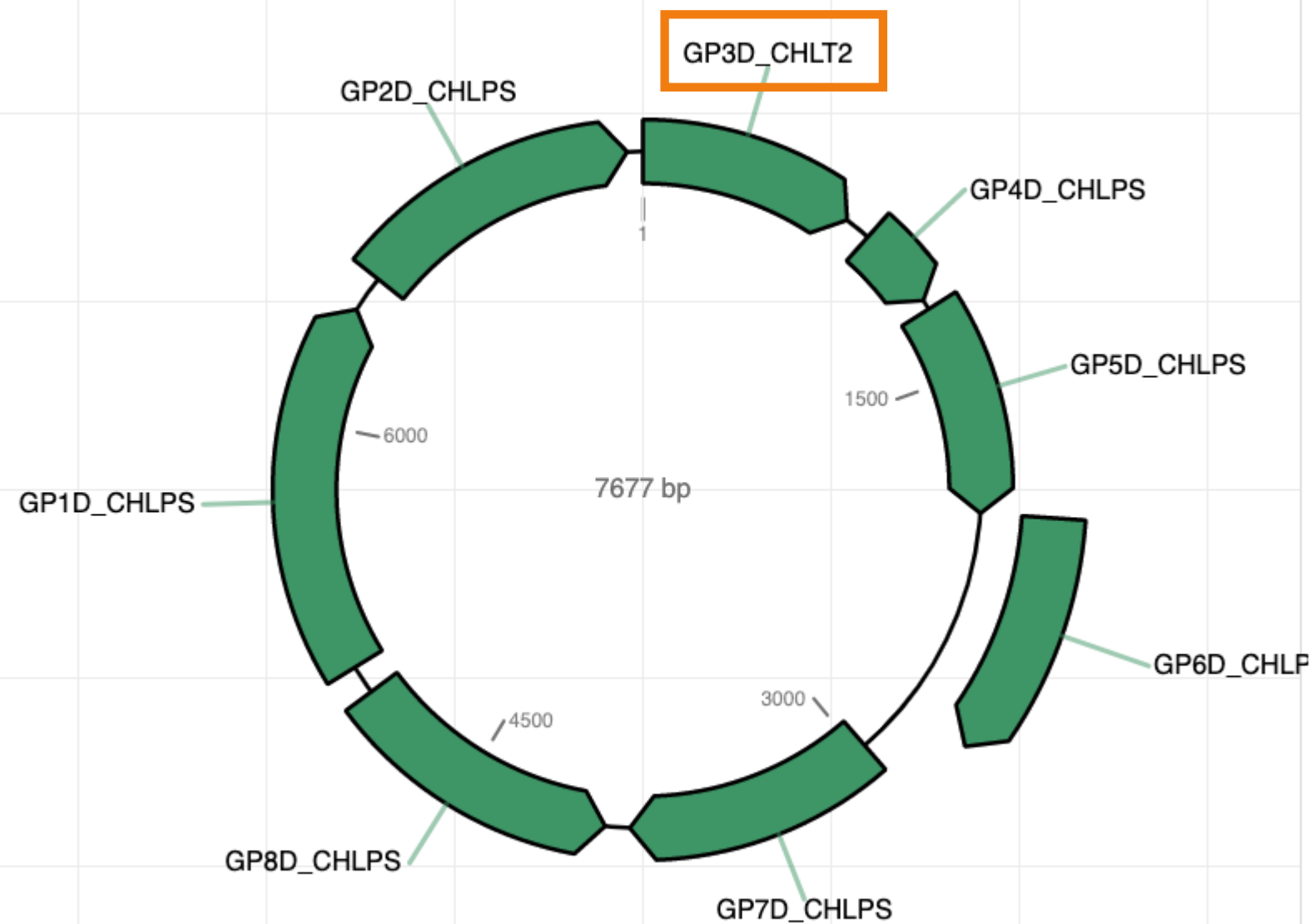

■ CDS

*C. psittaci* MN, re-assembled, re-arranged

Supplement: Supplementary file 8 — Additional file 8: Figure S8. Here, we exemplarily show the results of our re-assembly and re-arrangement efforts for the plasmid of C. psittaci strain MN. A) CDS annotation achieved with pLannotate for plasmid sequence directly obtained from NCBI. Note that genes GP5D_CHLPS and GP2D_CHLPS were only found with 68 and 52 % sequence similarity, respectively, which is marked by white arrows. B) Re-assembled plasmid sequence using corresponding raw-read data of strain MN and after re-arrangement using GP3D_CHLT2 as marker gene (orange frame). In the re-assembled plasmid, GP5D_CHLPS and GP2D_CHLPS achieved a sequence similarity of 99 and 100 %, respectively. Further details and results for all other plasmids can be found in the OSF (https://osf.io/rbca9). [file 12864_2023_9370_MOESM8_ESM.pdf]
